# Cucurbit[*n*]uril (*n* = 6, 7) Based Carbon-Gold Hybrids with Peroxidase-Like Activity

**DOI:** 10.3390/nano8050273

**Published:** 2018-04-24

**Authors:** Liangfeng Zhang, Yan Zeng, Simin Liu, Feng Liang

**Affiliations:** The State Key Laboratory of Refractories and Metallurgy, Coal Conversion and New Carbon Materials Hubei Key Laboratory, School of Chemistry & Chemical Engineering, Wuhan University of Science and Technology, Wuhan 430081, China; wustzlf@163.com (L.Z.); zengyan@wust.edu.cn (Y.Z.); liusimin@wust.edu.cn (S.L.)

**Keywords:** cucurbit[n]uril, gold nanoparticles, carbon-gold hybrid, peroxidase-like activity

## Abstract

Despite the combination of molecular recognition and local electric field enhancement endowing cucurbit[*n*]uril-capped metallic nanoparticles, indicating great potential in a variety of areas, prior work has paid little attention to carbonizing cucurbit[*n*]uril on the surface of gold nanoparticles, which may propose new carbon-gold hybrid materials with interesting applications. In this work, we developed a simple and cost-effective method to prepare carbon-gold hybrids by carbonizing cucurbit[*n*]uril modified gold nanoparticles. The as-prepared cucurbit[*n*]uril based carbon and carbon-gold hybrid materials have shown to possess peroxidase-like activity. All cucurbit[*n*]uril based nanomaterials exhibited high catalytic activity over a pH range 2–6 and more tolerant to high temperature (up to 60 °C) when compared to natural horseradish peroxidase.

## 1. Introduction

The cucurbit[n]uril (CB[n]) family, which bears a rigid hydrophobic cavity and two identical carbonyl fringed portals, is one of the most important macrocyclic containers in host-guest chemistry. The carbonyl oxygen atoms of the cucurbit[*n*]uril can coordinate directly to a variety of metal ions and lead to complicated nanoscale frameworks and architectures [1]. Since Li and colleagues unraveled the direct interactions between surface of gold nanoparticles and the electron-rich carbonyl rims of CB[n] for the first time [2], CB[n] has been commonly used in capping gold and other metallic nanoparticles with applications, such as in catalysis, drug delivery, electrochemical analysis and surface-enhanced Raman spectroscopy (SERS) [3,4]. In spite of this, there are very few reports of carbonization of CB[n] on the surface of gold nanoparticles, which may be expected to bring new carbon-gold hybrid materials with interesting applications. Previous studies have shown that carbon-gold nanocomposites have excellent biocompatibility, electric conductivity, and physical and chemical stability [5,6,7,8,9,10].

**Scheme 1:**
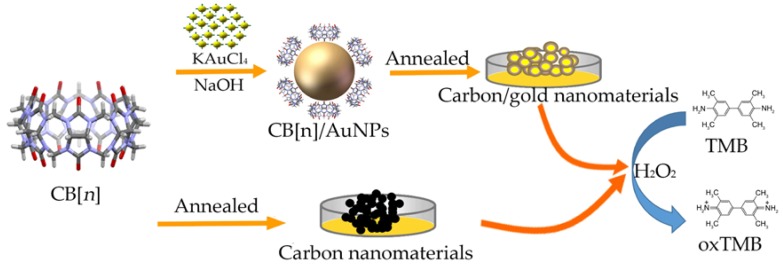
Schematic illustration of preparation of cucurbit[*n*]uril based carbon-gold and carbon nanomaterials with peroxidase-like activity.

The long-term goal of our group is to construct carbon, metal and carbon-metal hybrid nanomaterials for emerging catalytic, sensing and biological applications [11,12,13,14,15,16,17]. In our previous work, we developed carbon nanomaterials decorated with metal nanoparticles by ion implantation and chemical deposition [18,19,20], in which the metal particles adhered poorly to the carbon material. Recently, we developed a sonicationand solvothermal method to prepare carbon nanomaterials [21,22,23] that can degrade hydrogen peroxide to produce hydroxyl radicals, and thus possess intrinsic peroxidase-like activity [22]. Herein, we demonstrate a simple and cost-effective method to prepare carbon-gold hybrids by carbonizing cucurbit[n]uril modified gold nanoparticles, as illustrated in Scheme 1. The prepared materials exhibited high catalytic activity over a pH range 2~6 and were more tolerant to high temperature when compared to natural horseradish peroxidase (HRP).

## 2. Materials and Methods

### 2.1. Chemicals

Sodium hydroxide, hydrogen peroxide, disodium hydrogen phosphate dodecahydrate, sodium chloride and 3,3′,5,5′-tetramethylbenzidine (TMB) were purchased from Sinopharm Chemical Reagent Co., Ltd. (Shanghai, China). Horseradish peroxidase was purchased from Hubei Xinxin Belle Biotechnology Co., Ltd. (Wuhan, China). Potassium tetrachloroaurate (III) was purchased from Sahn Chemical Technology Co., Ltd. (Shanghai, China). Cucurbit[6]uril and cucurbit[7]uril were synthesized by our group [14,17].

### 2.2. Cucurbit[n]uril Directed Synthesis of Gold Nanoparticles (AuNPs)

The aqueous solution of CB[6] (5 mmol, 25 mL) and CB[7] (5 mmol, 25 mL) was prepared with the assistance of sodium chloride (29 mg) because CB[6] partially dissolved in double distilled water. After that, one equivalent amount of aqueous KAuCl_4_ (5 mmol, 25 mL) was mixed with CB[6] or CB[7] solution at room temperature. A yellow precipitate was immediately obtained, then an aqueous solution of NaOH (40 mmol, 50 mL) was added. The yellow precipitate disappeared, and a clear solution was obtained; then, a wine-red colored solution formed after 48 h, indicating the formation of the AuNPs.

### 2.3. Preparation of Cucurbit[n]uril Based Carbon-Gold and Carbon Nanomaterials

Initially, the resulting wine red AuNPs was washed three times with double distilled water by means of centrifugation. After that, AuNPs solution was sealed in Teflon-lined stainless-steel autoclave (100 mL) and reacted at 180 °C for 24 h by using Vacuum oven DZF-6020 (Shanghai Jinghong, Shanghai, China). After the autoclave was cooled down to the room temperature, the pre-carbonized materials were collected by means of centrifugation. The obtained khaki powder was dried in vacuum at 40 °C, then carbonized in quartz crucible up to 700 °C under a nitrogen atmosphere at a heating rate of 5 °C/min, and then held at that temperature for 1 h by using a vacuum tube furnace, OTF-1200X. Carbon nanomaterials were directly prepared from CB[6] and CB[7] in a quartz crucible by heating up to 700 °C under a nitrogen atmosphere at a rate of 5 °C/min, and then held at that temperature for 1 h.

### 2.4. Materials Characterization

The UV-vis absorption spectra were recorded on Shimadzu UV-vis-NIR spectrophotometer (Shimadzu, Tokyo, Japan). Transmission electron microscopy (TEM) was performed by using JEM-2100UHR STEM (JEOL, Tokyo, Japan). Energy-dispersive X-ray spectroscopy (EDS) measurements were performed with a spectrometer attached to PHILIPS XL30 TMP field-emission scanning electron microscopy (SEM) system (Philips, Hillsboro, OR, USA). The X-ray powder diffraction (XRD) pattern was tested on a X’Pert PRO MPD diffractometer (Panalytical B.V., Amsterdam, Holland) with nickel-filtered Cu K_α_radiation. A thermal gravimetric analyzer (TGA) STA449 (Nai Chi Scientific Instruments, Shanghai, China)was used for the investigation of the thermal properties of the samples.

### 2.5. Peroxidase-Like Activity

To investigate the peroxidase-like activity of carbon-gold and carbon nanomaterials, catalytic oxidation of the colorimetric substrate TMB in the presence of H_2_O_2_ was performed. In a typical experiment [22], the mixture of TMB (8 mM, 100 µL), carbon-gold (40 µL) or carbon nanomaterials (1 mg/mL), phosphate buffer (25 mM, 810 µL, pH = 4), together with H_2_O_2_ (1 M, 50 µL) are incubated at 35 °C for standard curve measurement. Under the above-mentioned condition, the catalytic reaction was incubated in the buffer solution under different pH (1.0–12.0) to investigate the pH effect on the tested reaction. Similarly, the reaction was incubated in the buffer solution under different temperature from 25 °C to 60 °C to examine the effect of temperature. HRP was investigated under the same conditions as the control. 

Under optimum conditions, the steady-state kinetic parameters of carbon-gold (C_CB[*n*]/AuNPs_) and carbon nanomaterials (C_CB[*n*]_) as catalysts were determined by using H_2_O_2_ and TMB as substrates. In the presence of H_2_O_2_ and TMB, the reaction was carried out at 35 °C in a 1 mL of a tube containing prepared nanomaterials or HRP in phosphate buffer solution (25 mM, pH = 4). The assays were carried out by varying concentration of TMB at a fixed concentration of H_2_O_2_ or varying concentration of H_2_O_2_ at a fixed concentration of TMB. The reaction kinetics measurements were performed by recording the absorbance reading at 652nm in the time scan mode.

## 3. Results and Discussion

### 3.1. Characterization of Prepared Nanomaterials

**Figure 1:**
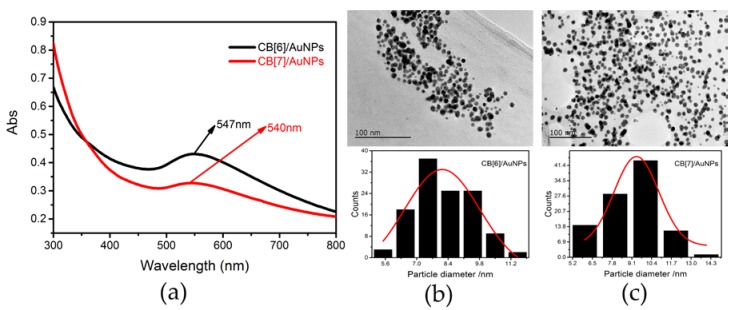
(**a**) UV-vis spectra of the prepared AuNPs. TEM micrographs and their corresponding histograms of (**b**) CB[6]/AuNPs and (**c**) CB[7]/AuNPs.

As illustrated in Scheme 1, KAuCl_4_ in CB[*n*] solution produced CB[n] capped AuNPs under a specific set of conditions [24,25,26,27]. The CB[*n*] plays the role of both reducing and protecting agents. The particles obtained were spherical in shape and had good dispensability as shown in Figure 1. UV-vis spectroscopy of AuNPs are shown in Figure 1a, the characteristic surface plasmon resonance band of these two kinds of AuNPs centered at 547 nm and 540 nm, respectively. The size of AuNPs was investigated by transmission electron microscopy (TEM), as shown in Figure 1b,c, and the mean diameter of the CB[n] capped AuNPs, CB[6]/AuNPs and CB[7]/AuNPs were 8.0 ± 1.5 nm and 10.0 ± 1.1 nm. 

**Figure 2:**
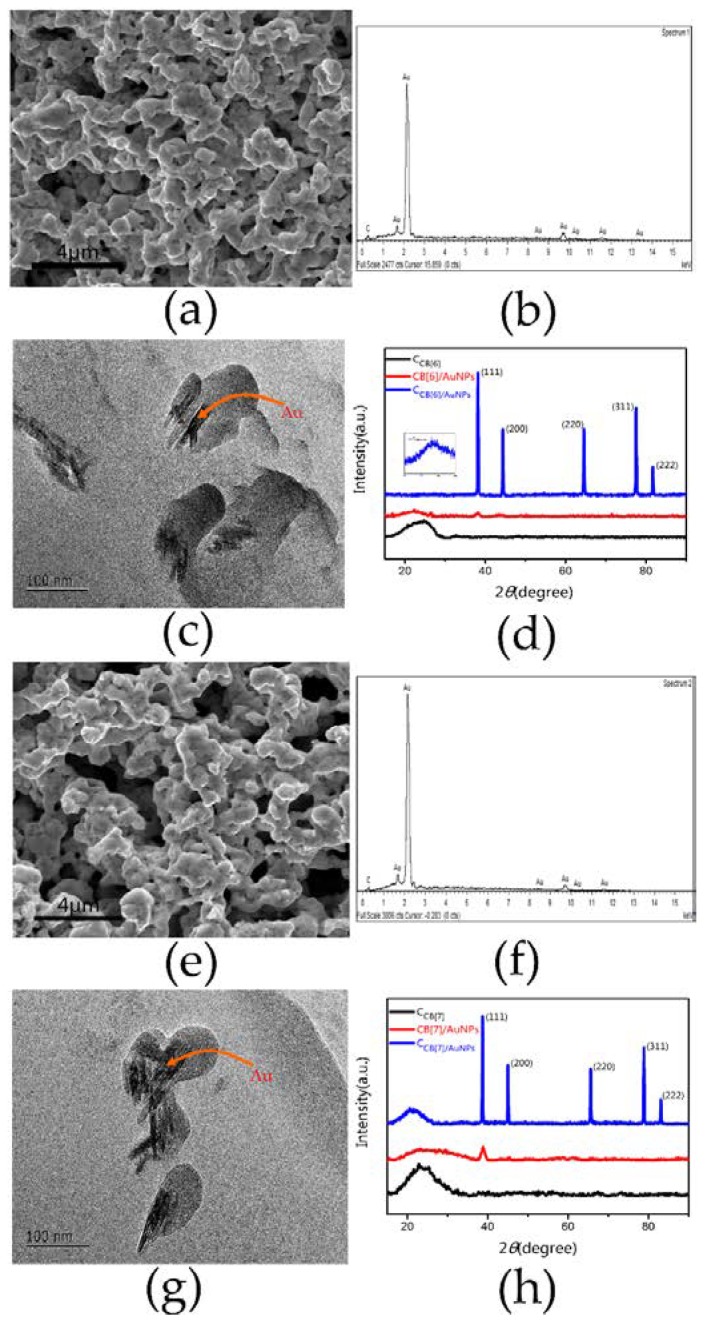
(**a**) Secondary electron SEM image of C_CB[6]/AuNPs_; (**b**) EDS pattern of C_CB[6]/AuNPs_; (**c**) TEM image of C_CB[6]/AuNPs_; (**d**) XRD pattern of CB[6] and corresponding materials; (**e**) Secondary electron SEM image of C_CB[7]/AuNPs_; (**f**) EDS pattern of C_CB[7]/AuNPs_; (**g**) TEM image of C_CB[7]/AuNPs_; (**h**) XRD pattern of CB[7] and corresponding materials.

In order to confirm the morphology of the annealed CB[6]/AuNPs and CB[7]/AuNPs hybrid materials (C_CB[6]/AuNPs_ and C_CB[7]/AuNPs_), scanning electron microscopy (SEM) and transmission electron microscopy (TEM) were applied to study the prepared materials. The SEM images in Figure 2a,e show that these hybrid materials have three-dimensional porous structures.EDS spectroscopy was further used to confirm the hybrid nanomaterials. As shown in Figure 2b,f, EDS only detected gold and carbon in the composites, suggesting that these hybrids are composed of gold and carbon elements. Moreover, *X*-ray diffraction patterns were utilized to detect the crystal structures of two hybrids. The diffraction peaks observed from the blue line of Figure 2d,h at 38.2°, 44.4°, 64.7° 77.7° and 81.8° could be ascribed to the (111), (200), (220), (311) and (222) planes of the Au crystals, and a broad peak at 20°–30° related to the amorphous carbon of cucurbit[*n*]uril [28,29], which further confirms that the hybrid materials are composites of gold and carbon. In addition, the black strip of Au is embedded in the carbon film irregularly, as evidenced by the TEM images in Figure 2c,g.

**Figure 3:**
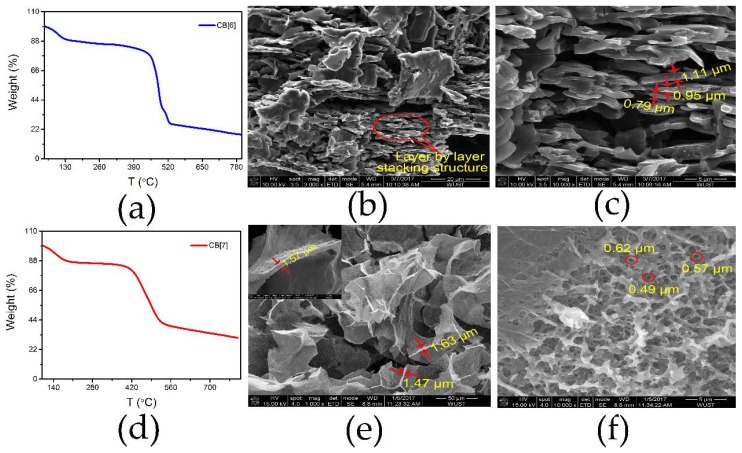
TGA curves of (**a**) CB[6] and (**d**) CB[7]; Secondary electron SEM images of C_CB[6]_: (**b**,**c**); Secondary electron SEM images of C_CB[7]_: (**e**,**f**).

Thermal gravimetric analysis (TGA) was used to investigate the weight loss of CB[6] and CB[7]. As shown in Figure 3, there are two steps to weight loss. The slight weight loss from the first stage could be attributed to the release of physically absorbed water, and the other loss is from the pyrogenation of cucurbit[n]urils [17]. The annealed cucurbit[7]uril (C_CB[7]_) is multilayer stacking of porous structures (the pore size is around 0.56 ± 0.13 μm), while the annealed cucurbit[6]uril (C_CB[6]_) is the layer by layer stacking structure, with no obvious pores detected. The thickness of C_CB[7]_ sheet (1.47 ± 0.29 μm) is thicker than that of C_CB[6]_ (0.97 ± 0.32 μm).

### 3.2. Peroxidase-Like Activity

**Figure 4:**
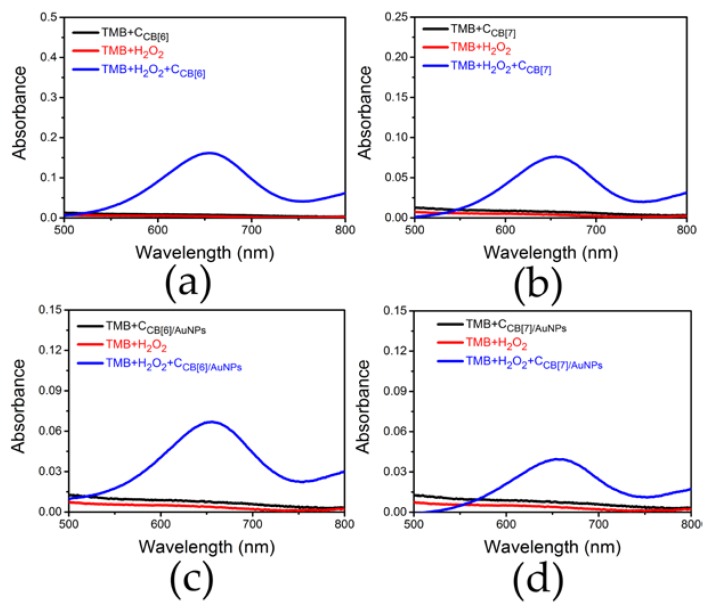
UV-vis spectra of different TMB system with (**a**) C_CB[6]_; (**b**)C_CB[7]_; (**c**) C_CB[6]/AuNPs_; (**d**) C_CB[7]/AuNPs_.

The catalytic performance of as-prepared carbon-gold and carbon nanomaterials were tested by using the peroxidase substrate TMB. Upon the addition of H_2_O_2_, the catalytic reaction can be monitored by detecting TMB absorbance change at 652 nm [22,30,31]. As shown in Figure 4, no obvious absorbance peak at 652 nm could be detected in the mixed solution of TMB and prepared materials of TMB and H_2_O_2_. After the addition of H_2_O_2_, the absorption peak at 652 nm was significantly enhanced in the presence of prepared nanomaterials over the same period. These results clearly demonstrated that prepared hybrids and carbon nanomaterials in this work possessed intrinsic peroxidase-like activity. As shown in Figure S1, with the increasing concentration of TMB, the absorbance at 652 nm changed quickly, suggesting that more TMB was oxidized. Furthermore, TMB can be detected by the prepared materials as low as 1 × 10^−4^ mol/L, which is similar to HRP [32,33,34,35]. 

**Figure 5:**
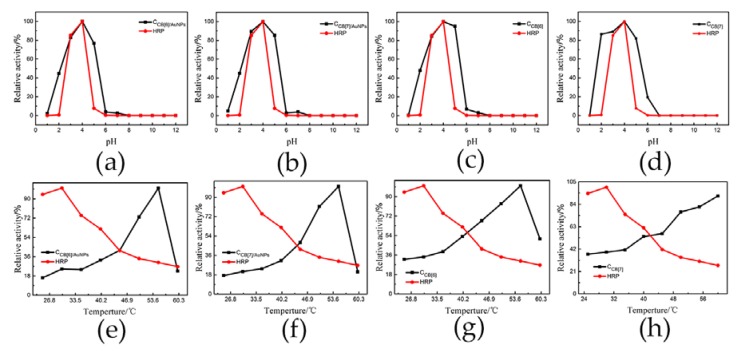
pH dependent response curves of different materials in this work and HRP: (**a**) C_CB[6]/AuNPs_; (**b**) C_CB[7]/AuNPs_; (**c**) C_CB[6]_; (**d**) C_CB[7]_. Temperature dependent response curves of different materials in this work and HRP: (**e**) C_CB[6]/AuNPs_; (**f**) C_CB[7]/AuNPs_; (**g**) C_CB[6]_; (**h**) C_CB[7]_.

Similar to other peroxidase mimics as well as the natural peroxidase HRP, the catalytic activity of hybrids and carbon nanomaterials is also dependent on pH and temperature [32,33]. It is well known that pH is an important parameter for a catalytic reaction. The pH dependent experiments were performed in the buffer solution with different pH values range from 1.0 to 12.0. Similar to the natural peroxidase HRP, the optimal pH is 4. In addition, hybrids and carbon nanomaterials are much more stable over a relatively wide pH range. As shown in Figure 5a–d, when the pH of the buffer solution is 5, the peroxidase-like activity of hybrids and carbon nanomaterials remained 80%, while the HRP almost drifted to inactive performance. Moreover, the influence of temperature on the TMB oxidation reaction catalyzed by the as-prepared nanomaterials and HRP were investigated by varying the reaction temperature from 25 °C to 60 °C, as illustrated in Figure 5e,f. Due to the sensitivity of HRP to temperature, the natural enzyme easily becomes denatured, while the catalytic activity of hybrids and carbon nanomaterials increased gradually with increasing experimental temperatures. However, the catalytic activity of hybrids and C_CB[6]_ dramatically decreased at a temperature higher than 55 °C, except for C_CB[7]_, which continuously increased with the higher temperature. This might be attributed to the honeycomb-like porous structure and larger pore volume of C_CB[7]_ as shown in Figure 3. Although it has been reported that Fe_3_O_4_@C nanoparticles [33] and CeO_2_ nanoparticles [34] exhibited temperature-dependent catalytic activity, the exact mechanism underlying such activity is still unclear. The evolution of oxidation of TMB under H_2_O_2_ as a function of pH and temperature was conducted. As shown in Figure S2, H_2_O_2_ could be more efficient in oxidizing TMB at higher temperatures under acidic conditions. Increasing the temperature could accelerate the decomposition of H_2_O_2_ to produce oxygen and benefit the oxidation of TMB. 

**Figure 6:**
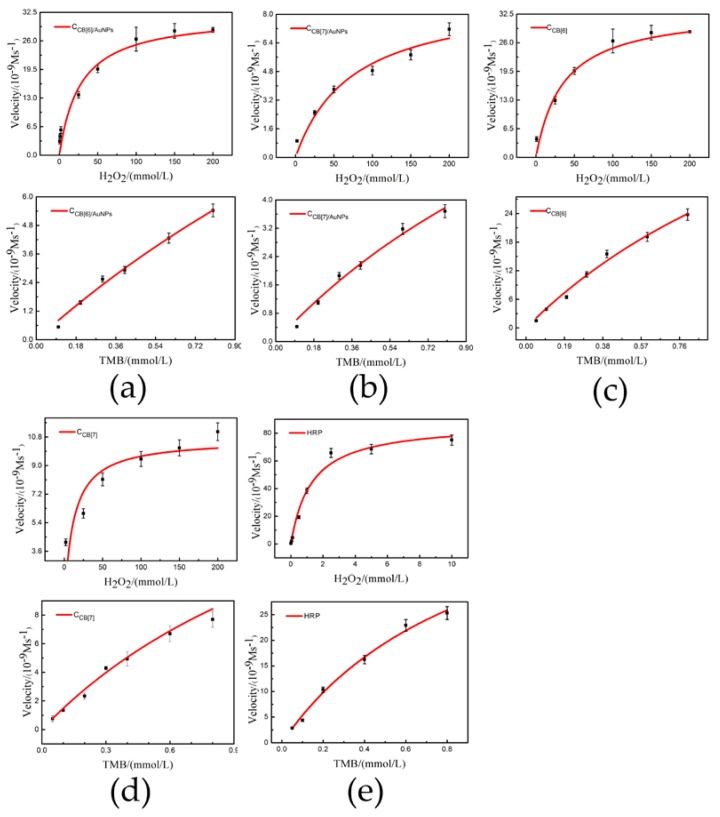
Steady-state kinetic curves of different materials in this work and HRP: (**a**) C_CB[6]/AuNPs_; (**b**) C_CB[7]/AuNPs_; (**c**) C_CB[6]_; (**d**) C_CB[7]_; (**e**) HRP.

For further analyzing the catalytic mechanism of hybrids and carbon nanomaterials, steady-state kinetics with TMB and H_2_O_2_ as substrates were investigated. A series of experiments were performed by varying the concentration of one substrate while fixing another as constant. Typical Michaelis-Menten curves (Figure 6) were received in a certain concentration of H_2_O_2_ or TMB. Maximum initial velocity (*V*_max_) and Michaelis-Menten constant (*K*_m_) were obtained using Lineweaver-Burk plot and were presented in Table 1. Typically, the lower *K*_m_ represents a higher enzyme-like affinity between the catalyst and the substrate [32,35]. The *K*_m_ value of hybrids with TMB was lower than that of carbon nanomaterials and higher than that of CB[n] capped AuNPs (Figure S3). This suggests higher binding affinity of hybrids to TMB than that of carbon nanomaterials, and lower binding affinity of hybrids to TMB than that of CB[*n*] capped AuNPs. Similarly, the *K*_m_ value of hybrids with H_2_O_2_ is slightly lower than that of carbon nanomaterials and higher than that of CB[*n*] capped AuNPs. The results can be attributed to the synergistic effect of gold and carbon, so hybrids with gold could absorb TMB and H_2_O_2_ more efficiently than carbon materials. However, the *V*_max_ of carbon nanomaterials is higher than that of hybrids. This is possible as the dispersion of hybrids in water is worse than that of carbon nanomaterials.

Similar to previous studies [36,37], the unsupported CB[*n*] capped AuNPs could catalyze the oxidation of TMB in the presence of H_2_O_2_. Typically, AuNPs could absorb H_2_O_2_ [38] and CB[n] on the gold tend to attract amino groups of TMB electrostatically [3,39], which likely to result in stronger affinity to reaction substrates (Table 1). After carbonization, the unique porous carbon architecture was produced, and the gold was embedded in the porous carbon aggregates, which possibly could benefit electron transfer [40]. Thus far, the hybrids in our case exhibited comparable peroxidase-like activity with Fe_3_O_4_, Pt, Pd and CuS nanoparticles, and much better binding affinity towards H_2_O_2_ (Table 1). There is still great potential to improve their ability as nano-enzymes by tuning compositions.

## 4. Conclusions

In the present work, we prepared well-dispersed AuNPs using cucurbit[n]uril (*n* = 6, 7) as reducing and protecting agents according to a facile and eco-friendly one-step synthesis. CB[n] based carbon-gold hybrids and carbon nanomaterials were then successfully prepared by carbonization, which is a simple, cost-effective, quick and practical approach. All CB[n]based nanomaterials possess intrinsic peroxidase-like activity, and the hybrids could absorb substrates more efficiently with the incorporated gold. This method guided constructing novel and enhanced carbon-gold multifunctional nanomaterials. Once guest molecules containing nitrogen, oxygen and other heteroatoms are included in the cavity of CB[n] on metallic nanoparticles, heteroatom-doped carbon-metal hybrids can be prepared.

## Figures and Tables

**Table 1:** Michaelis-Menten constant (*K*_m_) and maximum reaction rate (*V*_max_) of different nanomaterials in this work and HRP.

Catalyst	Substrate	*K*_m_ (mM)	*V*_max_ (10^−9^ M/s)
C_CB[6]_	TMB	2.4 ± 0.1	98.2 ± 3.8
	H_2_O_2_	2.2 ± 0.2	16.4 ± 1.8
C_CB[7]_	TMB	2.5 ± 0.1	34.7 ± 2.9
	H_2_O_2_	1.4 ± 0.1	5.2 ± 0.8
CB[6]/AuNPs	TMB	0.098 ± 0.008	10.1 ± 0.8
	H_2_O_2_	0.2 ± 0.07	3.0 ± 0.2
CB[7]/AuNPs	TMB	0.038 ± 0.003	10.2 ± 0.2
	H_2_O_2_	0.097 ± 0.02	3.9 ± 0.1
C_CB[6]/AuNPs_	TMB	1.9 ± 0.2	11.4 ± 1.2
	H_2_O_2_	1.3 ± 0.2	10.8 ± 1.5
C_CB[7]/AuNPs_	TMB	0.63 ± 0.12	6.4 ± 1.0
	H_2_O_2_	0.74 ± 0.08	1.7 ± 1.1
HRP	TMB	0.81 ± 0.11	47.6 ± 2.3
	H_2_O_2_	9.8 ± 0.04	87.6 ± 2.0
Fe_3_O_4_ magnetic nanoparticles [30]	TMB	0.098	34.4
	H_2_O_2_	154	97.8
Glutathione-capped Pd nanoparticles [41]	TMB	0.068	315
	H_2_O_2_	156	408
Glutathione-capped Pt nanoparticles [42]	TMB	0.079	328
	H_2_O_2_	73.6	302
Montmorillonite-supported CuS nanoparticles [43]	TMB	0.021	2.8
	H_2_O_2_	2.3	0.97

## Supplementary Materials

The following are available online at http://www.mdpi.com/2079-4991/8/5/273/s1, Figure S1: The time-dependent absorbance changes at 652 nm under different TMB concentration; Figure S2: (a) Temperature and (b) pH dependence for TMB and H_2_O_2_ system in the absence of nanomaterials; Figure S3: UV-vis spectra of different TMB system with (a) CB[6]/AuNPs and (b) CB[7]/AuNPs, and steady-state kinetic curves of (c) CB[6]/AuNPs and (d) CB[7]/AuNPs.
