# Supplementary material for: Cucurbit[n]uril (n = 6, 7) Based Carbon-Gold Hybrids with Peroxidase-Like Activity
Source: Nanomaterials (Basel). 2018 Apr 24;8(5):273. doi: 10.3390/nano8050273 (PMC5977287; doi:10.3390/nano8050273)
Supplement: Supplementary file 1 [file nanomaterials-08-00273-s001.pdf]

Supplementary Materials: Cucurbit[n]uril (n = 6, 7) Based Carbon-gold Hybrids with Peroxidase-like Activity

Liangfeng Zhang, Yan Zeng, Simin Liu and Feng Liang*

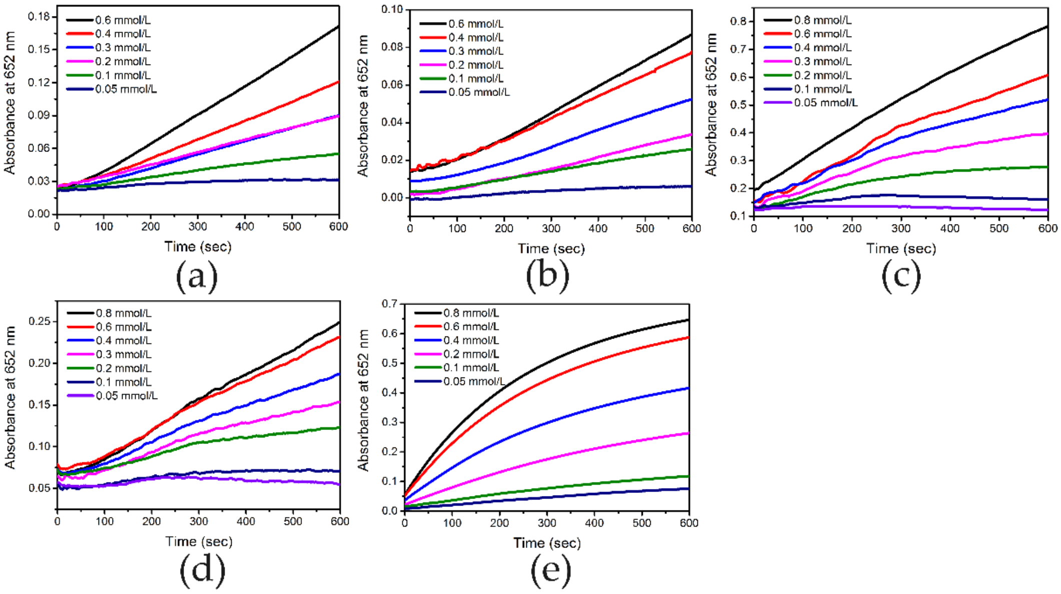

Figure S1. The time-dependent absorbance changes at 652 nm under different TMB concentration (25 mM Na_2HPO_4, pH 4.0) at 35 °C in the presence of fixed H_2O_2 and (a) $C_{CB[6]/AuNPs}$; (b) $C_{CB[7]/AuNPs}$; (c) $C_{CB[6]}$; (d) $C_{CB[7]}$; (e) HRP.

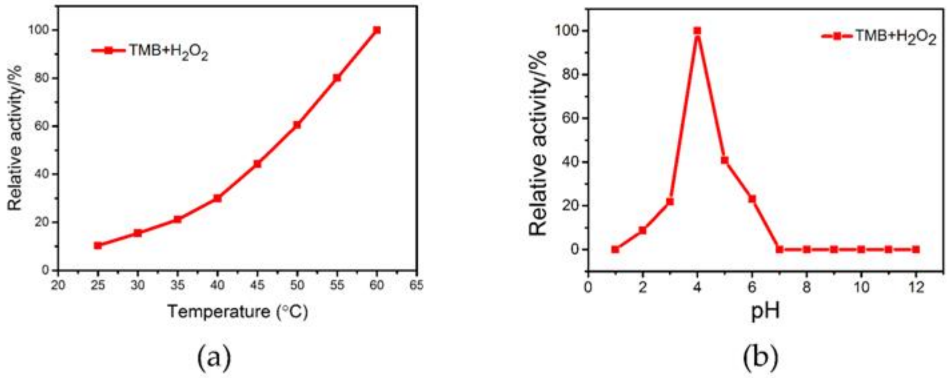

Figure S2. (a) Temperature and (b) pH dependence for TMB and H_2O_2 system in the absence of nanomaterials.

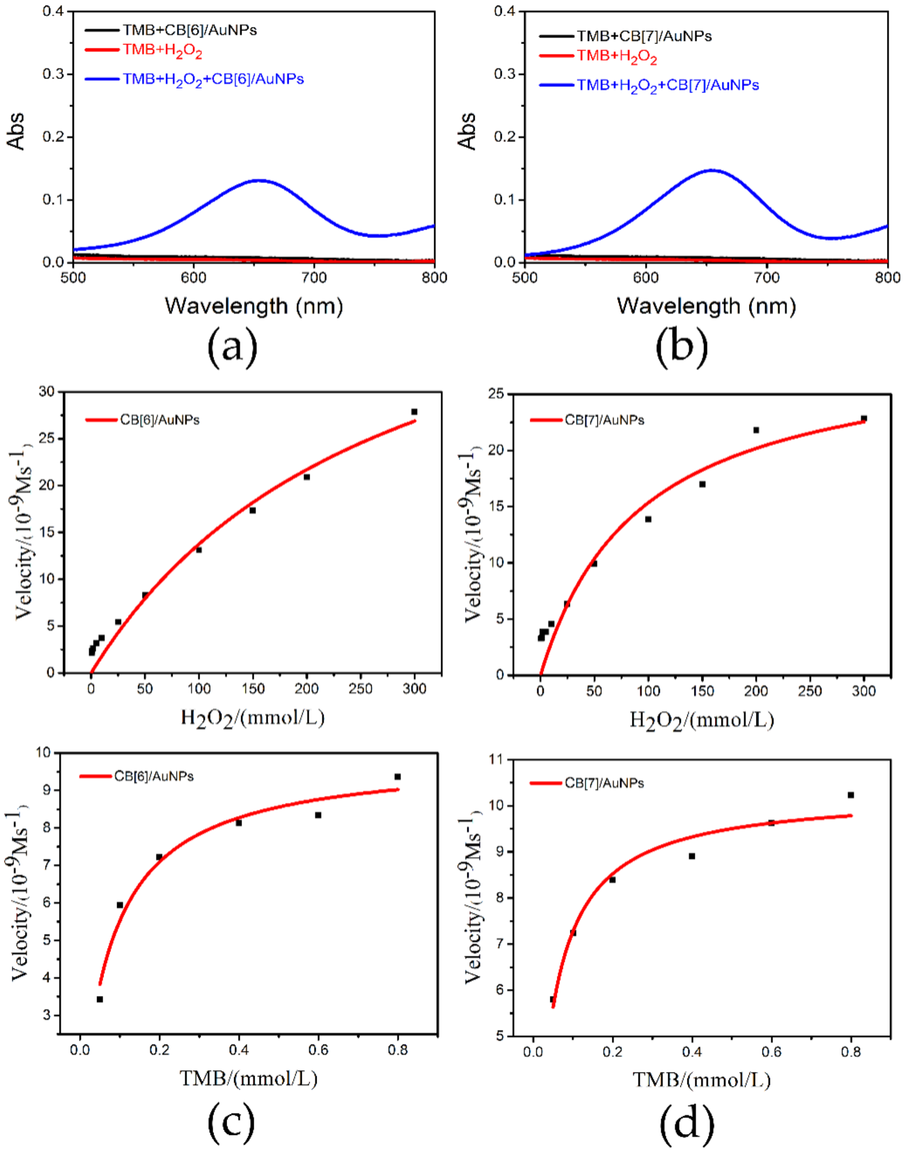

Figure S3. UV-vis spectra of different TMB system with (a) CB[6]/AuNPs and (b)CB[7]/AuNPs, and steady-state kinetic curves of (c) CB[6]/AuNPs and (d)CB[7]/AuNPs.

References:

1. Tao, Y.; Lin, Y.; Huang, Z.; Ren, J.; Qu, X. Incorporating Graphene Oxide and Gold Nanoclusters: A Synergistic Catalyst with Surprisingly High Peroxidase-Like Activity Over a Broad pH Range and Its Application for Cancer Cell Detection. *Adv. Mater*. **2013**, *25*, 2594-2599.

2. Song, Y.; Qu, K.; Zhao, C.; Ren, J.; Qu, X. Graphene Oxide: Intrinsic Peroxidase Catalytic Activity and Its Application to Glucose Detection. *Adv. Mater*. **2010**, *22*, 2206-2210.
